# Full Spectrum of LPS Activation in Alveolar Macrophages of Healthy Volunteers by Whole Transcriptomic Profiling

**DOI:** 10.1371/journal.pone.0159329

**Published:** 2016-07-19

**Authors:** Miguel Pinilla-Vera, Zeyu Xiong, Yutong Zhao, Jing Zhao, Michael P. Donahoe, Suchitra Barge, William T. Horne, Jay K. Kolls, Bryan J. McVerry, Anastasiya Birukova, Robert M. Tighe, W. Michael Foster, John Hollingsworth, Anuradha Ray, Rama Mallampalli, Prabir Ray, Janet S. Lee

**Affiliations:** 1 Department of Medicine, Division of Pulmonary, Allergy, and Critical Care Medicine, University of Pittsburgh, Pittsburgh, Pennsylvania, United States of America; 2 Department of Pediatrics, University of Pittsburgh, Pittsburgh, Pennsylvania, United States of America; 3 Department of Medicine, Duke University, Durham, North Carolina, United States of America; 4 Department of Medicine, Division of Pulmonary and Critical Care Medicine, Ohio State University, Columbus, Ohio, United States of America; 5 The Medical Specialty Service Line, Veterans Affairs Pittsburgh Healthcare System, Pittsburgh, Pennsylvania, United States of America; 6 Vascular Medicine Institute, University of Pittsburgh, Pittsburgh, Pennsylvania, United States of America; University of Tennessee Health Science Center, UNITED STATES

## Abstract

Despite recent advances in understanding macrophage activation, little is known regarding how human alveolar macrophages in health calibrate its transcriptional response to canonical TLR4 activation. In this study, we examined the full spectrum of LPS activation and determined whether the transcriptomic profile of human alveolar macrophages is distinguished by a TIR-domain-containing adapter-inducing interferon-β (TRIF)-dominant type I interferon signature. Bronchoalveolar lavage macrophages were obtained from healthy volunteers, stimulated in the presence or absence of ultrapure LPS in vitro, and whole transcriptomic profiling was performed by RNA sequencing (RNA-Seq). LPS induced a robust type I interferon transcriptional response and Ingenuity Pathway Analysis predicted interferon regulatory factor (*IRF)7* as the top upstream regulator of 89 known gene targets. Ubiquitin-specific peptidase (USP)-18, a negative regulator of interferon α/β responses, was among the top up-regulated genes in addition to *IL10* and *USP41*, a novel gene with no known biological function but with high sequence homology to *USP18*. We determined whether *IRF-7* and *USP-18* can influence downstream macrophage effector cytokine production such as IL-10. We show that IRF-7 siRNA knockdown enhanced LPS-induced IL-10 production in human monocyte-derived macrophages, and USP-18 overexpression attenuated LPS-induced production of IL-10 in RAW264.7 cells. Quantitative PCR confirmed upregulation of *USP18*, *USP41*, *IL10*, and *IRF7*. An independent cohort confirmed LPS induction of *USP41* and *IL10* genes. These results suggest that IRF-7 and predicted downstream target USP18, both elements of a type I interferon gene signature identified by RNA-Seq, may serve to fine-tune early cytokine response by calibrating IL-10 production in human alveolar macrophages.

## Introduction

Alveolar macrophages perform a central function in maintaining lung homeostasis and orchestrate the response to injury and repair. Effective surveillance requires the ability to robustly respond to stimuli by rapidly triggering a transcriptional network of innate immune signals. The sentinel function of alveolar macrophages also requires the ability to exquisitely calibrate transcriptional responses. Recent findings indicate the plasticity of macrophages in response to diverse stimuli [1]. While sensing of pathogen associated molecular patterns (PAMPs) by macrophages involves the induction of genes that activate innate immunity [1], less is known about the regulatory measures that are also activated within the transcriptional program. Moreover, what constitutes a “healthy” macrophage response to PAMP activation is unclear, as much of the focus in human lung studies has been centered on dysregulated pathways in disease compared to a control population [2–5]. This approach has been useful, but does not fully explore the possibility that alveolar macrophages in health exert tight control over innate immune activation at the initiation of LPS responses by inducing a number of regulatory genes to calibrate and prevent over-amplification of the inflammatory cascade yet, at the same time, optimize the production of effector molecules for anti-microbial host defense.

TLR4 polymorphisms that confer “LPS hypo-responsiveness” in humans [6] are associated with increased susceptibility to gram negative septic shock but protection from atherogenesis [7, 8]. Recipients of lung allografts bearing TLR4 polymorphisms that confer “LPS hypo-responsiveness” are protected from acute cellular rejection compared with wildtype individuals[9]. This finding is independent of the donor tissue polymorphism status, implicating recipient immune cells rather than donor parenchymal cells as potential drivers of this phenomenon. As recipient alveolar macrophages quickly repopulate the airspaces following allograft transplantation, others have suggested that innate activation of recipient alveolar macrophages may contribute to the development of acute rejection [9]. The precise mechanisms for these genetic associations are not known, as the full spectrum of the LPS activation response in human alveolar macrophages is incompletely understood. Still others have shown that LPS recognition by TLR4 induces activation through the adaptor protein TIR-domain-containing adapter-inducing interferon-β (TRIF; TICAM1) and downstream type I interferon signaling [10]. Type I interferons can exert a complex, often dual role comprised of both stimulatory as well as immune regulatory function. Activation of type I interferon response may serve to optimally calibrate both positive and negative regulatory signals required to eliminate offending agents on the one hand, yet simultaneously prevent excessive inflammation and injury on the other hand. We aimed to explore the full spectrum of the LPS transcriptional program in human alveolar macrophages of healthy volunteers, focusing upon IRF-7, a master regulator of the type I interferon pathway, and its relationship to early immune-regulatory cytokine production. We hypothesized that LPS induces IRF-7 to calibrate key regulatory genes such as IL-10 known to be involved in the modulation of the inflammatory response.

## Methods

### Subjects

Healthy, nonsmoking volunteer subjects between the ages of 18–65 were recruited for this study. We recruited consecutive healthy volunteers who met the following inclusion criteria: (1) age between 18–65 years; (2) good general health with no personal history of lung disease; (3) non-smoker; (4) able to provide written informed consent and participate in bronchoscopy. Exclusion criteria included: (1) age less than 18 years; (2) have clinically significant illness; (3) respiratory tract infection within 4 weeks of participation; (4) receipt of an investigational product or device, or participation in a drug research study within the prior month; (5) active smoking history; (6) known or suspected hypersensitivity or allergy to medications used during bronchoscopy; (7) any condition that, in the investigator’s opinion, places the subject at undue risk for complications from bronchoscopy with bronchoalveolar lavage. All participants signed informed consent to undergo fiberoptic bronchoscopy and to have samples collected used for analysis. The University of Pittsburgh Institutional Review Board approved the study.

### Fiberoptic Bronchoscopy and Preparation of Cell Suspensions

A standardized protocol was used to perform fiberoptic bronchoscopy and bronchoalveolar lavage (BAL) was obtained from the right middle lobe using 200 mL sterile 0.9% NaCl instilled in 50 mL aliquots. All BAL samples were immediately processed under sterile conditions. The cells were recovered from BAL fluid by centrifugation (400*g* for 15 minutes at 4°C) and the supernatant was collected, aliquoted into equal volumes, and stored at -80°C for later use. If necessary, red blood cells were removed from the BAL cell pellet by brief hypotonic lysis using molecular biology degree nuclease free and endotoxin free deionized water. BAL total cell counts and differentials were performed from cytospins that were prepared using Diff-Quick stain set. BAL cells were resuspended in PBS with 2% FBS for flow cytometric analysis.

### Flow cytometry and macrophages immunolabeling

Alveolar macrophages were labeled with one or more monoclonal anti-human fluorochrome conjugated antibodies, or respective isotype controls: anti-human CD16 (PE-Cy7, BD Biosciences, Cat#557744; isotype control mouse IgG1κ), anti-human CD14 (APC, Beckman, Cat#IM2580U; isotype control mouse IgG2α), anti-human CD14 (FITC, Beckman, Cat#IM0645U; isotype control mouse IgG2α), anti-human HLA-DR (PE, Beckman, Cat#IM1639U; isotype control mouse IgG1), anti-human CD15 (Alexa fluor 647, BD Biosciences, Cat#562369; isotype control mouse IgG1κ), anti-human CCR2 (Alexa fluor 647, BD Biosciences, Cat#561744; isotype control mouse IgG2b), anti-human CCR5 (FITC, BD Biosciences, Cat#561747; isotype control mouse IgG2α, κ), anti-human CXCR3 (PE, BD Biosciences, Cat#55063; isotype control mouse IgG1κ), anti-human CD36 (APC, BD Biosciences, Cat#561822, isotype control mouse IgM, κ), anti-human CD68 (BD Biosciences, Cat#556078; isotype control mouse IgG2bκ), anti-human CD91 (FITC, BD Biosciences, Cat#550496; isotype control mouse IgG1κ), anti-human CD163 (BD Biosciences, Cat#556018, isotype control Mouse IgG1κ), anti-human CX_3_CR1 (MBL, Cat# D070-4, isotype control Rat IgG2b, κ).

The cells were incubated with a mixture of the above fluorochrome-conjugated antibodies at manufacturer suggested concentrations or concentrations determined by titration in a total volume of 100 μL in cold PBS + 2% FBS shielded from light at 4°C for 30 minutes. Human FcR blocker was used to block Fc-receptors prior to immunostaining. Following the initial wash with cold PBS + 2% FBS, cells were fixed with 2% paraformaldehyde in PBS + 2% FBS. Flow cytometry data were acquired on BD bioscience FACS LSRII cell analyzer. Compensation and data analysis were performed with FACSDiva version 6.2 and FCS express version 3. Cells that were large with high granularity were gated out from debris by examining events on side scatter and forward scatter window. Autofluorescent^hi^ cells, representing macrophages, were gated in FITC and PE. Surface antigen expression was determined by the mean fluorescent intensity (MFI) ratio, defined as (MFI_antigen_-MFI_control antibody_/ MFI_control antibody_)[11].

### Sample preparation and analysis of RNA-Seq data

Total RNA was extracted by miRNeasy Mini Kit (Qiagen) and was used as starting material for deep sequencing using the Illumina TruSeq RNA Sample Preparation v2 Guide. As described above, a standardized protocol was used to obtain BAL from the right middle lobe of subjects undergoing fiberoptic bronchoscopy using 200 mL sterile 0.9% NaCL instilled in 50 mL aliquots. Cells were centrifuged (400*g* for 15 minutes at 4°C), supernatant was collected, and BAL total cell counts and differentials were performed from cytospins. BAL samples from five of the healthy volunteers (samples 11–15, Table 1) were cultured with RPMI1640 + 10% human AB serum and supplemented with 1% Antibiotic-Antimycotic (Life Technologies), with or without ultrapure LPS 100 ng/ml (*E*. *coli* 0111:B4, List Biological Laboratories, Cat# 421, Lot# 4217A1) at 37°C and 5% CO_2_ for 6 hours. Ultrapure LPS was chosen to minimize possibility of contamination by other bacterial components [1]. Previous studies have used concentrations of LPS ranging from 10 ng/ml-1000 ng/ml [12–14]. The cell culture supernatants were collected and stored at -80°C for later use. Total RNA was extracted by miRNeasy Mini Kit (Qiagen). RNA concentrations were measured by Nano-Drop, and RNA integrity was checked using a bio-analyzer. Total RNA (1–4 μg) was used as starting material for deep sequencing using the Illumina TruSeq RNA Sample Preparation v2 Guide. Briefly, mRNA was purified with oligo-dT beads, fragmented with magnesium and heat-catalyzed hydrolysis, and used as a template for first- and second-strand cDNA synthesis with random primers. The cDNA 3’ ends were adenylated, followed by adaptor ligation and a 15-cycle PCR to enrich DNA fragments. Quantification of cDNA libraries was performed by using Kapa Biosystems primer premix kit with Illumina-compatible DNA primers. For cluster generation, the TruSeq SR Cluster Kit v2-cBot-GA was used, and cDNA libraries were loaded onto the flow cell at a final concentration of 8 pM. Single-read sequencing was performed on the Illumina Genome Analyzer II. RNA sequence data were uploaded to Geospiza Genesifter for analysis (Geospiza, Inc., Seattle, WA).

**Table 1:** Demographics of healthy volunteers and bronchoalveolar lavage cell counts.

Sample	Gender	Age	Cell Count (× 10^4^/ml)	Macrophage %	PMN %	Lymphocyte %
1	M	21	4.08	99.5	0.5	0.0
2	F	24	5.34	100.0	0.0	0.0
3	F	20	10.04	78.6	21.4	0.0
4	M	58	3.82	99.2	0.8	0.0
5	M	37	5.94	98.3	0.0	1.7
6	F	25	2.91	97.5	0.6	1.9
7	F	43	8.47	86.7	3.3	10.0
8	M	29	5.8	98.3	1.7	0.0
9	M	56	7.54	96.3	0.4	3.3
10	F	33	3.18	98.3	1.3	0.4
11	M	35	10.67	100.0	0.0	0.0
12	F	22	6.44	96.0	0.8	3.3
13	F	20	6.44	99.2	0.0	0.8
14	M	31	2.89	100.0	0.0	0.0
15	F	38	6.89	98.2	0.0	1.8

BAL samples from consecutive healthy volunteers who met the inclusion and exclusion criteria were included and no samples were excluded in the cell count and differential analysis. Gender, age, cell counts and differentials are depicted. Immunophenotyping by flow cytometric analysis was conducted with cells obtained from the first ten consecutive volunteers 1–10. Whole transcriptomic profiling was conducted by RNA-Seq following in vitro stimulation in the presence or absence of ultrapure LPS using cells obtained from the subsequent consecutive healthy volunteers 11–15.

### Quantitative RT-PCR

Real time quantitative PCR was performed according to Applied Biosystem 7900HT Fast Real-Time PCR System manufacturer instructions for the following human genes: *CXCL11* (Hs04187682_g1), *CXCL10* (Hs01124251_g1), *IL10* (Hs00961622_m1), *USP41* (Hs02596851_gH), *USP18* (Hs00276441_m1), *IRF7* (Hs01014809_g1); *ADAMTS15* (Hs00373520_m1), *HPGD* (Hs00960586_g1), *LRP1* (Hs00233856_m1), and *CD36* (Hs01567185_m1). *18S* or *GADPH* were used as housekeeping genes as indicated in the respective figures. For each sample, relative expression values were normalized to the housekeeping gene using the ΔΔ Ct method. For experiments performed in RAW264.7 cells as detailed below, real time quantitative PCR was performed using the following mouse *il10* primers: Forward 5’- TGGGAAGAGAAACCAGGGAGA-3’; Reverse 5’-GTTTTCAGGGATGAAGCGGC-3’.

### Human Monocytes Derived Macrophages Isolation and siRNA transfection

Blood was obtained from human volunteers by venipuncture of an antecubital vein using sterile technique following informed consent. These volunteers were separate from the volunteers for the fiberoptic bronchoscopy studies. The University of Pittsburgh Institutional Review Board approved the study. Briefly, blood was anticoagulated using citrate phosphate dextrose solution. Approximately 30 ml were laid on Ficoll-Paque Plus for gradient centrifugation and the mononuclear cell layer at the interface was harvested. Mononuclear cells were washed three times with PBS, then resuspended in RPMI 1640, and plated onto petri-dishes. The cells were incubated at 37°C, 5% CO2 for 1 hour. Plated cells were then washed with RPMI 1640 containing fresh media with 10% autologous serum plus recombinant human-M-CSF at 50 ng/ml. Cells were incubated for 5 days with one round of media change. Following differentiation, Human Monocyte Derived Macrophages (HMDM) were detached from the petri-dishes by placing the dishes on ice for 1 hour. The cells were counted, aliquoted and centrifuged down at 400*g* for 5 minutes. The cells were then resuspended in 100 μl of Amaxa Human Macrophage Nucleofector^®^ Solution containing 800 nM of control siRNA or IRF-7 siRNA and transferred to a cuvette. The cells were transfected by electroporation in Nucleofector I device with program Y-10 (Lonza, Walkersville, MD). Cells were retrieved by rinsing the cuvette with 500 μl of Aim-V medium with 10% autologous serum and transferred to 12-well plates for incubation. Twenty-four hours later, the media was changed and HMDM were exposed to ultrapure LPS (*E*. *coli* 0111:B4, 100 ng/ml, List Biological Laboratories, Cat# 421, Lot# 4217A1) for 24 hours. The cell culture supernatants were harvested for cytokine analysis by ELISA (Duoset, R&D systems) and the cells were lysed in Trizol for RNA extraction or lysed with complete protein lysis buffer for immunoblotting. RNA was extracted using Qiagen miRNeasy mini kit and cDNA was synthesized using Invitrogen SuperScript III Reverse Transcriptase kit. Real time quantitative PCR was performed according to Applied Biosystems 7900HT Fast Real-Time PCR System manufacturer instructions.

### RAW264.7 cell culture and transfection

RAW264.7 cells were obtained from ATCC and cultured in Dulbecco’s Modified Eagle’s Medium (DMEM) (ATCC, cat# 30–2002) with 10% FBS and Penicillin-Streptomycin (Sigma, cat# P4333) as specified by the manufacturer. The cells were transfected with empty vector or USP-18-V5 (2 μg) for 48 hours. The cells were then incubated in the presence of LPS (*E*. *coli* 0127:B8, 200 ng/ml, Sigma, Cat# L4516) or vehicle for 24 hours and then harvested for real time quantitative PCR, immunoblotting, or ELISA.

### USP-18-V5 plasmid preparation

The RAW264.7 cells were cultured as detailed above. Total RNA was extracted from human macrophages and cDNA was synthetized by using reverse transcriptase (Biorad). *USP18* cDNA was synthetized and amplified by PCR with the following primers: *USP18* cDNA Forward: 5’-CACCATGAGCAAGGCGTTTGGG-3’ and *USP18* cDNA Reverse: 5’-GCACTCCATCTTCATGTAAACCA-3’. The *USP18* cDNA was purified and inserted into pcDNA3.1D/V5-His TOPO vector (Thermo Fisher Scientific). The cDNA was confirmed by DNA sequencing.

### Immunoblotting

HMDM cells were cultured and treated as detailed above. Cells were lysed using lysis buffer containing complete protease inhibitors (cOmplete mini, Roche). The samples were loaded and ran using NuPAGE SDS-PAGE electrophoresis system (4–12%, Invitrogen) and transferred to a nitrocellulose membrane for 1 hour at 34 volts and 4°C. The membrane was blocked using 4% milk in Tris-buffered saline, 0.1% Tween 20 (TBST) and probed for 16 hours with rabbit anti-IRF-7 antibody (Abcam, MA, Cat#ab109255) (1/1000 in TBST at 4°C). After washing, the membrane was probed with secondary anti-rabbit IgG antibody (1/2000 in TSBT at room temperature (Cell Signaling Technology, Inc, MA Cat #7074S). The membrane was developed using SuperSignal West Pico Chemiluminescent Substrate (Thermo) and exposed using Kodak image station 440CF for 10 minutes. Rabbit anti-tubulin antibody (1/1000 4% milk-TBST) was used as loading control.

For RAW264.7 cells, they were cultured as detailed above. The cells were washed with cold PBS and lysed with lysis buffer containing 20mM Tris-HCl (pH 7.4), 150 mM NaCl, 2mM EGTA, 5 mM β-glycerophosphate, 1 mM MgCl_2_, 1% Triton X-100, 1mM sodium orthovanadate, 10 μg/ml of protease inhibitors, and 1 μg/ml pepstatin. Approximately 20 μg of protein were subjected to SDS-PAGE, electrotransferred to membranes and immunoblotted with antibodies to V5 tag (Santa Cruz) and β-actin (Sigma).

### Independent Cohort Validation

To confirm specific gene transcripts, additional real time PCR was performed at Duke University on alveolar macrophages from an independent cohort of subjects undergoing bronchoscopy through a Duke University Institutional Review Board approved protocol exploring the effects of human responses to ozone exposure. Of the 54 subjects who were enrolled in this study, 24 subject samples who had undergone filtered air exposure (control) were selected at random for validation of candidate genes. The subject samples were selected on the basis of the subject’s completion of the entire experimental protocol, and sufficient quality and quantity of RNA to perform the analysis. The average age of subjects was between 20–34 years of age. Requirements for inclusion included: normal range of body mass index; no evidence of respiratory disease; and non-smoking status. As a part of the approved protocol, subjects underwent controlled exposure to filtered air with intermittent periods of controlled ambulation on a treadmill. On the day following filtered air exposure, participants underwent a flexible bronchoscopy with bronchoalveolar lavage. From the BAL, cells were counted and then cytospin analysis was performed to confirm percentage of cell components. From the BAL cells, alveolar macrophages were isolated; cultured in RPMI1640 medium supplemented with 10% heat-inactivated FBS, 100 units/ml penicillin, and 100 μg/ml streptomycin; and then plated in a 24 well-plate at a density of 200,000 cells per well. The cells were maintained in a CO_2_ incubator at 37°C for 2 hours, after which, the media was replaced to remove non-adherent cells. The cells then underwent stimulation with either saline (control) or LPS (100 ng/mL; Sigma) for 2 hours. Following stimulation, the supernatant was removed and the cells were harvested for RNA.

RNA extraction of macrophage cells was performed using a Qiagen RNeasy Mini Kit (4th edition, Valencia, CA). DNase treatment was performed using DNase I (Ambion, Austin, TX), followed by cDNA synthesis (BioRad). Real Time PCR was performed using an ABI SDS 7500 instrument (Applied Biosystems) with SYBR Green Reagent (Clontec Laboratories Inc., Mountain View, CA). The same primers sequences were used as in the primary cohort. Each individual subject had macrophages exposed to both saline and LPS to account for intra-subject variability. The data is normalized to an 18S RNA housekeeping gene and is reported as fold change over the matched control sample for each individual subjects alveolar macrophages.

### Statistical analysis

RNA-Seq data was analyzed with GeneSifter Anaylsis Edition (Geospiza, Inc, WA). A difference threshold of 1.5 was used for comparison between groups. The mapping quality threshold, used to reduce the effect of low intensity or poor quality reads, and performed by a built-in algorithm within the GeneSifter software, was set at 20 as recommended by the manufacturer. Gene expression data was log-transformed and analyzed using Student’s t-test. A Benjamini-Hochberg adjusted *p*-value < 0.05 was considered statistically significant. With this test, the output yielded 3279 differentially expressed genes between the two groups. A partition around medoids for clustering was performed, allowing for the division of this set of genes into an up-regulated cluster (670) and a down-regulated cluster (2609). Gene ontology (GO) analysis was performed with GeneSifter using z-scores, which were calculated with the following formula *z* = (*r*–*n R*/*N*)/√(n(*R*/*N*)(1 –*R*/*N*)(1 –(*n*– 1/*N*– 1)), where *r* = number of genes meeting selection criteria with the specified GO, n = total number of genes measured with the specific GO term, *R* = total number of genes meeting selection criteria, and *N* = total number of genes measured. z-score < -2 and > 2 was considered statically significant. These calculations were performed by the GeneSifter software with default settings and without input from the investigators. Additional molecular pathway design and network analysis was performed using the Ingenuity Pathway Analysis (QIAGEN, CA). For this analysis, the genes mean expression levels from the RNA-Seq data obtained from GeneSifter were uploaded into the Ingenuity Pathway Analysis software platform following the manufacturer’s instructions. Analysis of the uploaded raw data was performed by this software using default settings and without further intervention by the investigators. An overlap *p* value was calculated by the software to identify potential upstream regulators using built-in algorithms and settings. This *p* value measures whether there is significant overlap between the genes expression state in the dataset and the genes known to be regulated by a transcriptional regulator. This was considered significant if *p*-value < 0.01, with Fisher’s Exact test. The statistical analysis in the remaining figures was performed using GraphPad Prism 5 (GraphPad, La Jolla, CA). The specific statistical tests are detailed in each figure legend. A *p*-value < 0.05 using two tailed tests was considered statistically significant.

## Results

### Surface immunophenotyping of alveolar macrophage populations from healthy volunteers

**Fig 1:**
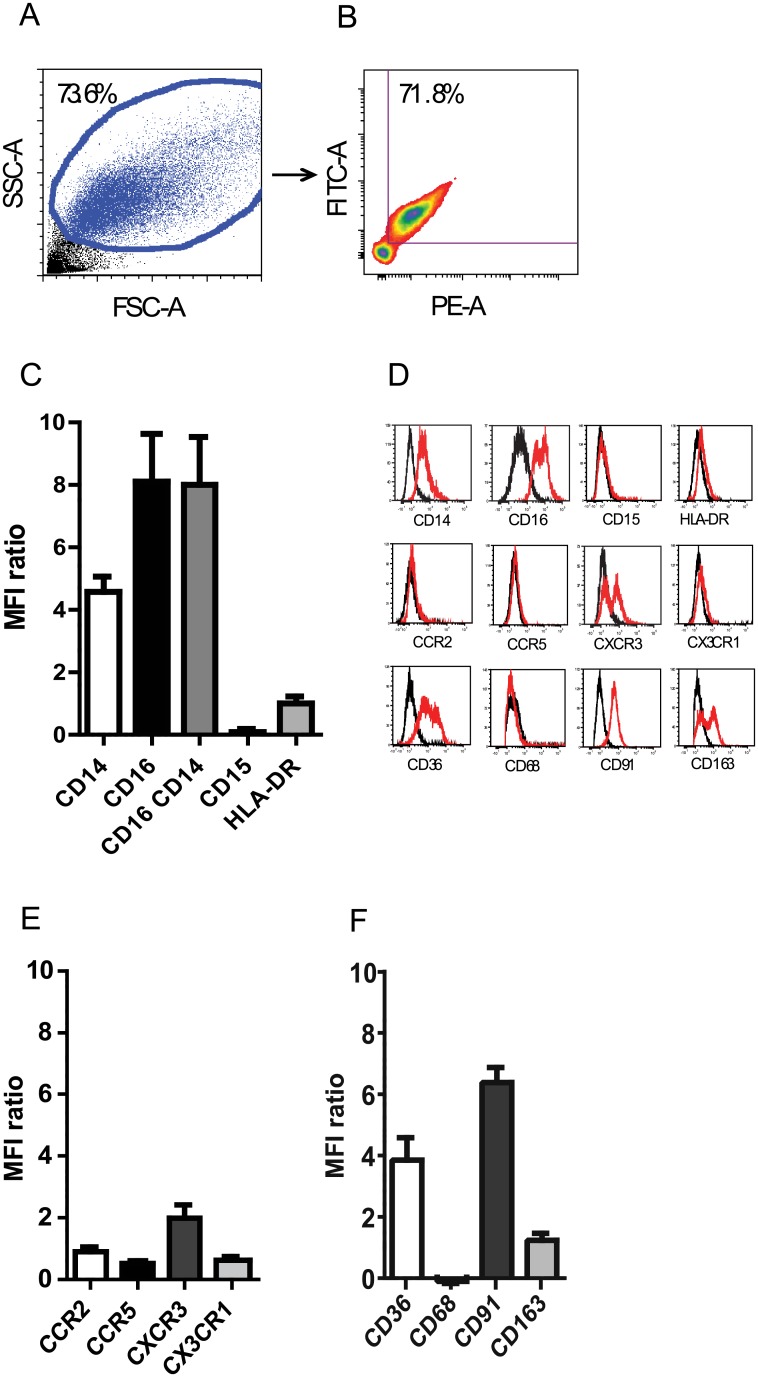
Surface immunophenotyping of BAL macrophages from healthy volunteers. (A) Alveolar macrophages obtained from the BAL of healthy volunteers were identified by size (FSC-A), granularity (SSC-A) and (B) autofluorescence. (C) Resident alveolar macrophage populations are CD14^+^/CD16^+^ and HLA-DR^lo^/CD15^neg^, as depicted by the mean fluorescence intensity (MFI) ratio where the MFI ratio = (MFI_antigen_-MFI_control antibody_)/ MFI_control antibody_[11]. (D) Histograms of surface antigens expressed by alveolar macrophages. Isotype control antibody staining is depicted in black. Antibodies to specific antigen immunostaining are depicted in red. (E) Alveolar macrophage surface expression of chemokine receptors CCR2, CCR5, CXCR3, and CX_3_CR1 as depicted by MFI ratio. (F) Alveolar macrophage surface expression of scavenger receptors CD36, CD68, CD91 and CD163 (n = 10).

Bronchoalveolar lavage (BAL) fluids from healthy, non-smoking volunteers were obtained for analysis. Subject gender, age, and BAL cell counts are shown (Table 1). The mean age was 32.8 years, 53.3% of participants were female and the average number of cells in the BALF was 6.03 x 10^4^ cells/ml. As expected, the majority of the cells in the airspaces were macrophages (96.4%) as assessed by cytospin analysis. Human macrophages are highly autofluorescent [15, 16]. This autofluorescent property facilitates isolation of macrophages by flow cytometry and BAL cells were confirmed to be large, granular cells by back-gating (Fig 1A and 1B). The majority of the granular cells are CD14^+^CD16^+^ indicating their myeloid/monocytic lineage and negative for CD15 (Fig 1C). Thus, resident macrophages were identified by granularity, size and auto-fluorescence and were *CD14*^*+*^*CD16*^*+*^ and *HLA-DR*^*lo*^*CD15*^*neg*^ (Fig 1C).

There is limited data on primary human alveolar macrophages from healthy volunteers. To determine the repertoire of surface receptors that characterizes human airspace macrophages under basal conditions, we focused upon two families of receptors, chemokine and scavenger receptors. Prior studies suggest that surface chemokine receptor expression such as CCR2 and CCR5 represent markers of innate immune activation or recently recruited monocytes and that resident alveolar macrophages in humans do not express CCR2 [17], CCR5 [18], or CX_3_CR1[19] under homeostatic conditions. Consistent with prior findings, alveolar macrophages minimally expressed CCR2, CCR5, or CX_3_CR1. However, a subset of these cells showed surface CXCR3 (Fig 1D and 1E) as previously reported [20]. Scavenger receptors are membrane receptors that bind and endocytose modified lipoproteins and represent a subclass of pattern recognition receptors involved in the recognition and phagocytosis of certain PAMPs and damaged cells [21]. Alveolar macrophages show surface expression of the scavenger receptors CD36, and CD163 (Fig 1D and 1F). In contrast, CD68, a pan-macrophage marker primarily found intracellularly, and localizing to lysosomes and endosomes, showed minimal surface expression [15]. CD91, low-density receptor-related protein (LRP)-1 or α2-macroglobulin receptor, involved in lipid homeostasis, clearance of apoptotic cells and sensor for necrotic cell death [22] through interaction with various ligands such as calreticulin [23, 24], and heat shock proteins gp96, hsp70, hsp90 [25, 26] is highly expressed by alveolar macrophages (Fig 1D and 1F). Thus, human airspace macrophages under homeostasis highly express scavenger receptors that are involved in the recognition of pathogen, modified self, or components of necrotic or apoptotic cells. Furthermore, these macrophages show minimal to low expression of CCR2, CCR5 and CX3CR1, providing support that these cells are not freshly recruited inflammatory monocytes.

### Transcriptomic analysis of human alveolar macrophages after LPS exposure shows a network of signaling pathways activation characterized by a prominent type I interferon response

**Fig 2:**
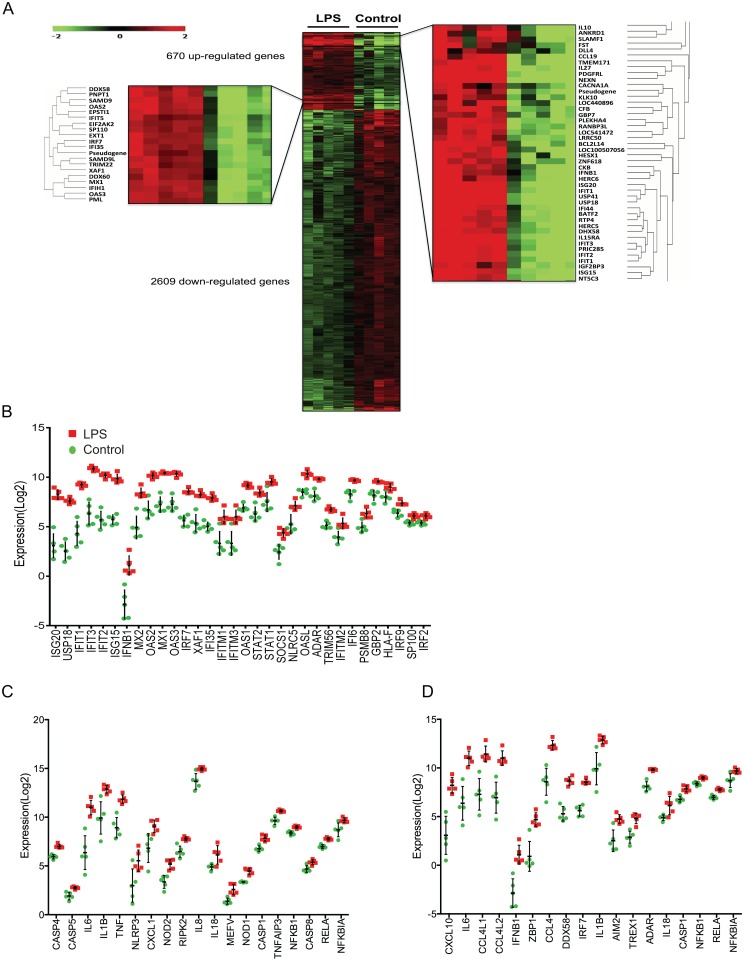
Heat Maps of differentially regulated genes and individually regulated genes within select gene pathways. RNA-Seq was performed in human alveolar macrophages from healthy volunteers to assess transcriptomic changes following ultrapure LPS stimulation in vitro (n = 5). (A) Of 3279 genes that were differentially expressed, 670 were upregulated and 2609 were downregulated and depicted in the heat map. Each column in the heat map represents a study subject and each row represents a gene. The order in the heat map was obtained in unbiased fashion by hierarchical clustering centered around genes with average linkage and Euclidean algorithm to establish the distance between genes. Further clustering analysis from the 670 upregulated genes was centered arbitrarily around genes of interest *IL10* (right) and *IRF7* (left) using the same clustering parameters as above. The distance between genes demonstrated in the dendogram is proportional to the similarity in the gene expression pattern between two genes. Color bar represents log_2_-fold changes in expression level. Student’s t-test, adjusted *p*-value < 0.05 with Benjamini-Hochberg correction was used to determine the 3279 differentially expressed genes. A partition around medoids was performed for clustering the genes into upregulated or downregulated state. (B) Differences in RNA expression level (log_2_) between control and LPS for upregulated genes assigned to the top Gene Ontology term *response to type I interferon* are depicted in descending order from left to right. (C) Differences in RNA expression level (log_2_) between control and LPS for upregulated genes in the top KEGG terms *NOD-like receptor pathway* and (D) *cytosolic DNA sensing pathway* are depicted in descending order from left to right. Each point depicts the log_2_-transformed gene expression value of an individual. Errors bars represent SD of the mean for each group.

RNA-Seq was performed on BAL cells from healthy volunteers following *in vitro* stimulation with ultrapure LPS (*E*. *coli* 0111:B4). BAL cells from the same subjects in the absence of LPS served as controls. There were 670 upregulated genes and 2609 downregulated genes (Fig 2A). As expected, upregulated genes include *MYD88* following TLR4 activation and downstream induction of TRAF family member-associated NF-kappa-B activator (TANK)-binding kinase 1 *(TBK1)*, *NF-κB* and cytokines *IL6*, *TNF*, *IL1B*, *IL12A*, *IL12B*, *CXCL10*, *and CXCL11*. Gene ontology (GO) enrichment analysis showed that *response to type I interferon* was the top upregulated term (z-score 19.21) after *cytokine mediated signaling pathway* (z-score 19.22, Table 2). Consistent with the GO enrichment analysis, the top up-regulated terms identified in the KEGG pathway analysis were *Influenza A* and *measles* related pathways, representing the strong type I interferon gene signature observed in LPS-stimulated alveolar macrophages. Upon closer inspection of the type I interferon response, KEGG pathway analysis identified activation of a number of cytosolic PRRs including *NOD-like receptor* (z-score 10.39), *retinoid acid-inducible gene (RIG)-I like receptors* that sense RNA (z-score 8.37), and *stimulator of interferon genes (STING)*-dependent PRR pathways (z-score 9.83) that sense cytosolic DNA in addition to *NFκB* (z-score 10.58), *JAK-STAT* (z-score 7.53), and *toll-like receptor signaling pathway* (z-score 8.85) (Table 2). Fig 2B–2D shows the expression level differences between control and LPS for upregulated genes corresponding to the top GO or KEGG terms. In addition, the strong type I interferon signature was not driven by a single individual in our dataset. Both TREX1, a 3’→5’ DNA exonuclease and STING-dependent responses [27], and ADAR, an RNA-editing enzyme that converts adenosine to inosine in double stranded RNA [28], prevent the accumulation of cytosolic nucleic acids that can promote chronic activation of the inflammatory cascade and were among the up-regulated genes (Fig 2D). Down-regulated KEGG pathways include *aminoacyl-tRNA biosynthesis*, *ribosome biogenesis in eukaryotes*, *basal transcription factors*, *ubiquitin-mediated proteolysis* and *PPAR* signaling *pathway* (Table 3, S1 Table containing 100 top down-regulated genes).

**Table 2:** Gene ontology and KEGG reports of up-regulated gene pathways.

**Ontology: biological process**	**List**	**Gene Set**	**z-score**
Cytokine-mediated signaling pathway	58	211	19.22
Response to type I interferon	32	71	19.21
**KEGG Pathway**	**List**	**Gene Set**	**z-score**
NF-kappa B signaling pathway	24	92	10.58
NOD-like receptor signaling pathway	18	57	10.39
Cytosolic DNA-sensing pathway	18	62	9.83
Cytokine-cytokine receptor interaction	43	275	9.72
Toll-like receptor signaling pathway	22	102	8.85
RIG-I-like receptor signaling pathway	17	71	8.37
Jak-STAT signaling pathway	25	155	7.53

The List value is the number of affected genes from the gene set in the group. The Gene set value shows the total number of genes from the dataset that are in each gene ontology category. Z-score in the GO and KEGG reports above 2 indicates the term occurs more frequently than expected by chance.

**Table 3:** Gene Ontology and KEGG report of down-regulated gene pathways.

**Ontology: biological process**	**List**	**Gene Set**	**z-score**
Cellular metabolic process	1180	7474	9.98
Cellular macromolecule metabolic process	915	5558	9.5
Primary metabolic process	1185	7618	9.31
Metabolic process	1276	8458	8.49
**KEGG Pathway**	**List**	**Gene Set**	**z-score**
Aminoacyl-tRNA biosynthesis	20	41	7.01
Ribosome biogenesis in eukaryotes	28	77	6.33
Basal transcription factors	14	44	3.86
Ubiquitin mediated proteolysis	32	137	3.85
mRNA surveillance pathway	21	90	3.1
PPAR signaling pathway	17	71	2.91

The List value is the number of affected genes from the gene set within a pathway. The Gene set value shows the total number of genes from the dataset that are in each gene ontology category. The z-score in the GO and KEGG reports above 2 indicates the term occurs more frequently than expected by chance.

### Identification of novel Ubiquitin Specific Peptidase 41

LPS stimulated alveolar macrophages showed enrichment of interferon stimulated genes (ISGs) including *ISG15*, *IFIT1*, *IFIT2*, *IFIT3*, *MX1*, *MX2*, *OAS1*, *OAS2*, *OAS3*, *IFNB1* in addition to antimicrobial guanylate binding proteins *GBP2* (mouse homolog *Gbp1*), *GBP4* (mouse homolog *Gbp3*), *GBP5* (mouse homolog *Gbp5*), and *GBP7* (mouse homolog *Gbp7*) (Fig 2B, S2 Table). Immunoresponsive gene 1 (*IRG1*) encodes a protein with cis aconitate decarboxylase activity involved in itaconic acid production [29], and indoleamine 2,3-dioxygenase 1 (*IDO1*) is involved in tryptophan catabolism [30, 31]. It is interesting that IRG1 and IDO1 are two key metabolic enzymes highly induced in alveolar macrophages and previously implicated in antimicrobial host defense and endotoxin tolerance (S2 Table)[30, 31]. *USP18*, an inhibitor of type I interferon signaling [32–34] was also highly upregulated (Fig 2B, S2 Table), suggesting tight regulation of innate immune activation even at the onset of LPS-stimulated responses.

USPs are a diverse group of cysteine peptidases which deconjugate ubiquitin and ubiquitin-like proteins from their target substrates [35]. USP-18 is an interferon-induced USP, which deconjugates ISG-15, a ubiquitin-like modifier, from its substrate. However, independent of USP-18 isopeptidase activity, USP-18 negatively regulates type I interferon signaling *in vivo* by blocking JAK1 interaction with IFNAR2 [32]. Loss-of-function mutations in human intracellular *ISG15* leads to enhanced response to type I IFN rather than impaired anti-viral immunity, because in humans, intracellular ISG-15 stabilizes USP-18 and prevents USP-18 proteolysis and degradation by ubiquitination [33]. Notably, *USP41* is among the top five highly inducible genes that encodes a predicted cysteine peptidase with high sequence similarity to USP-18 near conserved regions that are essential for catalytic activity (S2 Table)[35]. In addition, as depicted in the heat map (Fig 2A) *USP41* is closely clustered to *USP18* and other type 1 interferon related genes suggesting similar expression pattern and biological activity. Based upon sequence alignment of human USPs, USP-41 is predicted to exhibit deubiquitylating activity given the presence of Cys, His, Asn residues that comprise the catalytic triad domain and surrounding conserved Cys-box, QQD box, and His box regions common to all USPs with enzymatic function [35]. However, its biological function remains unknown.

### Interferon Regulatory factor 7 is the top upstream regulator in the early response to LPS of human alveolar macrophages and regulates IL-10 expression

**Fig 3:**
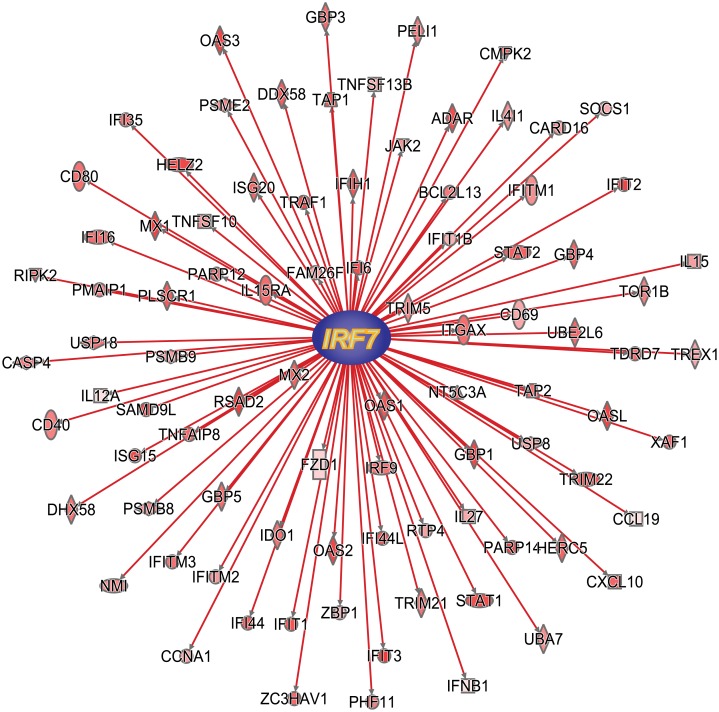
IRF-7 is predicted as a top upstream regulator of 89 known gene targets. Analysis of RNA-Seq dataset was performed with Ingenuity Pathway Analysis software, which predicted IRF-7 to be the top upstream regulator (overlap *p-value* = 3.5x10^-37^, Fisher’s Exact test, z-score 9.28, n = 5). The increased expression of *IRF7* is consistent with the increased expression state of 89 known target genes, predicted from the literature. Red lines connecting the upstream regulator and the target gene represent a positive consistent correlation. A red colored molecule represents the gene is upregulated in the dataset. More intense red color represents higher expression level state in the dataset.

**Fig 4:**
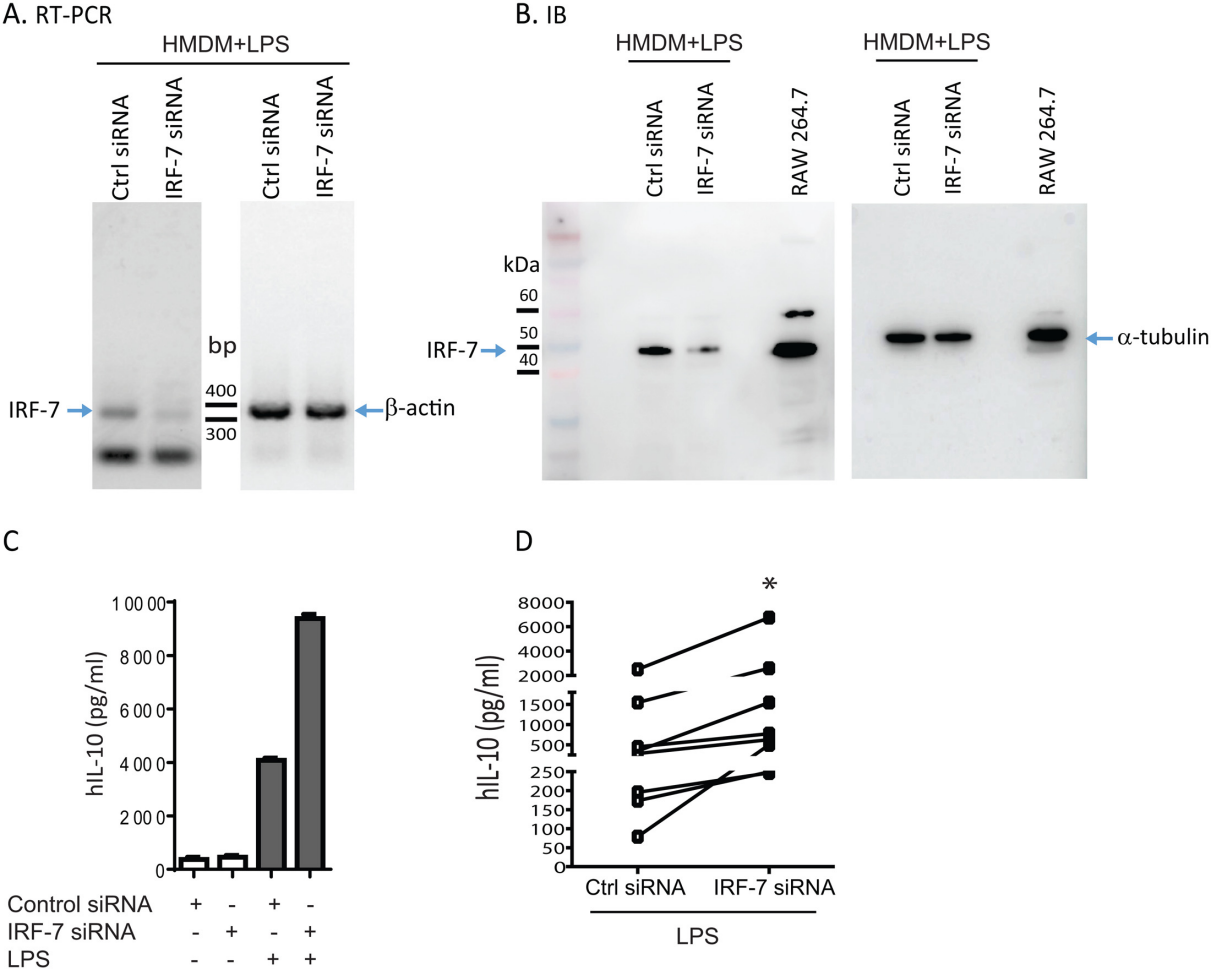
*IRF7* gene knockdown enhances LPS-induced human macrophage IL-10 production. Human monocyte derived macrophages were isolated from the peripheral blood of healthy volunteers and cultured for 5 days in RPMI 1640 media containing fresh media with 10% autologous serum and recombinant human-M-CSF (50 ng/ml) before transfection with control siRNA or IRF-7 siRNA. The cells were stimulated in the presence or absence of ultrapure LPS (100 ng/ml, *E*. *coli* 0111:B4) for 24 hours. (A) RT-PCR for IRF-7 was performed and confirmed gene knockdown with IRF-7 specific pooled siRNA as compared to control siRNA-treated cells after exposure to LPS (expected amplicon size IRF-7: 327 bp). β-actin gene was used as a house-keeping gene controlling for input sample quantity (expected amplicon size β-actin: 353 bp). (B) IRF-7 siRNA treated cells showed attenuated IRF-7 protein expression by immunoblotting, when compared to control siRNA treated cells in the presence of LPS (expected band size IRF-7 ~50 kDa). Alpha tubulin was used as loading control. Unstimulated RAW264.7 cell lysates were used as positive control for IRF-7. (C) Cell culture supernatants from HMDM were harvested for quantification of IL-10 production by ELISA. IRF-7 knockdown enhances IL-10 production in the presence of LPS. IL-10 production is minimal in the absence of LPS. (D) Composite of 8 independent experiments showing significant increase in IL-10 production by ELISA in supernatants of IRF-7 siRNA treated HMDMs compared to control siRNA in the presence of LPS. Each point represents matched samples, (*p-value < 0.05 Wilcoxon matched-pairs sign rank test, n = 8).

IRF-7 expression is inducible during type I interferon responses triggered by viral and bacterial PAMPs including LPS [36–38]. In human alveolar macrophages, IRF-7 expression is strongly induced after LPS stimulation (Fig 2A, left zoom-in heat map panel, Fig 2B, S2 Table). Ingenuity Pathway Analysis predicted IRF-7 as the top upstream regulator (overlap p-value = 3.5 x 10^−37^, z-score 9.28) of 89 known gene targets that include *STAT1*, *STAT2*, *IRF9* and *IFNB1* (Fig 3). Given the tight regulation of innate immune activation at the onset of LPS responses and previous reports showing the requirement for type I interferon signaling in LPS-induced production of the key immune-regulatory cytokine IL-10 [39], we wondered whether IRF-7 is involved in regulating LPS-induced IL-10 responses. This hypothesis was further supported by the close hierarchical clustering of *IL10* to other type I interferon related genes and known targets of *IRF7* in the heat map (Fig 2A, right zoom-in heat map panel). In addition, *IL10* is among the top 10% of up-regulated genes (S2 Table) in our dataset. IL-10 limits auto-inflammatory responses [40], but over-vigorous IL-10 production early during bacterial infection can be detrimental to the host by suppressing inflammation necessary to clear bacteria [41–43]. HMDMs were isolated from healthy donors to assess the role of IRF-7 in LPS-driven IL-10 production. *IRF7* knockdown by siRNA was confirmed by RT-PCR and immunoblotting (Fig 4A and 4B). Surprisingly, inhibition of *IRF7* by siRNA increased LPS-induced IL-10 production in HMDMs (Fig 4C and 4D), indicating that *IRF7* induction serves to curtail early IL-10 response.

**Fig 5:**
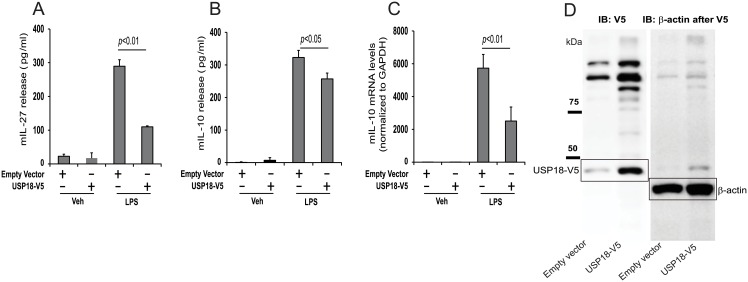
USP-18 overexpression in RAW264.7 cells decreases production of IL-27 and IL-10. RAW264.7 cells were transfected with empty vector or USP-18-V5 (2 μg) for 48 hours. The cells were then incubated in the presence of LPS (200 ng/ml) or vehicle for 24 hours. Cell supernatants were assessed using ELISA showing (A) decrease in production of mouse IL-27 in USP-18-V5 transfected cells compared to empty vector in the presence of LPS and (B) decreased levels of mouse IL-10 in USP-18-V5 transfected cells after LPS stimulation. RNA was extracted from the RAW264.7 transfected cells and (C) real time quantitative PCR was performed showing decreased IL-10 RNA levels in the USP-18-V5 transfected cells compared to control vector in the presence of LPS. (D) Immunoblotting was performed in the RAW264.7 cells transfected with empty vector or USP-18-V5 (expected band size ~45 kDa) and assayed for V5 tag protein amount. β-actin detection was used as loading control.

*USP18* is a known target of IRF-7 [44, 45]. To determine whether USP-18 can influence LPS-induced immune-regulatory cytokine production, *USP18* cDNA was cloned from human alveolar macrophages and overexpressed in mouse RAW264.7 cells. As shown in Fig 3, IL-27 is a target of IRF-7 [44] and a pleotropic cytokine that can mediate type I interferon dependent anti-inflammatory effects by constraining Th17 development and inflammation [46]. Similar to *IL10*, *IL27* is among the top 10% of up-regulated genes and closely clustered with *IL10*, *USP18* and other type I interferon related genes (Fig 2A, right zoom-in heat map panel, S2 Table) in our dataset. When compared to empty vector transfected cells, USP-18 over-expression reduced both LPS-induced IL-27 and IL-10 protein release (Fig 5A and 5B) in this cell line. LPS-induced *IL10* gene expression was also attenuated in USP-18 overexpressing cells (Fig 5C). Together, these data support the concept that IRF-7 and USP18, both elements of a type I interferon gene signature identified by RNA-Seq, calibrates innate immune activation by restraining early IL-10 production.

### Confirmation of differentially regulated genes by quantitative real time PCR and validation in a separate cohort

**Fig 6:**
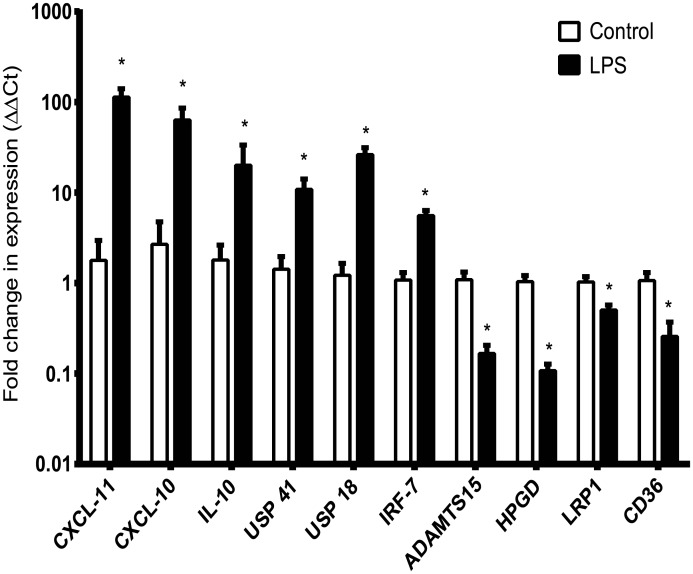
Validation of RNA-sequencing data by quantitative real-time PCR. Representative up-regulated and down-regulated target genes from the RNA-Seq data set were validated with real time quantitative PCR using the same samples used for RNA-Seq assays (*p<0.05 two-tailed t-test, vs. respective control, n = 4–5). Values represent mean (+/-) SEM of relative gene expression changes vs. 18S housekeeping gene, calculated by the ΔΔCt method. Human alveolar macrophages were stimulated in the presence or absence of LPS for 6 h.

**Fig 7:**
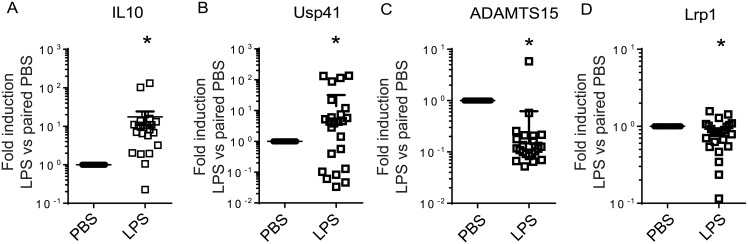
Expression profile of representative genes was validated in a second, independent cohort of BAL macrophages by real-time quantitative PCR. (*p<0.05 two-tailed paired t-test vs. respective control, n = 24). Values represent mean (+/-) SEM of relative gene expression changes vs. 18S housekeeping gene, calculated by the ΔΔCt method. Human alveolar macrophages in the second, independent cohort were stimulated in the presence or absence of LPS for 2 h.

Several genes with known or potential relevance for the innate immune response that were significantly upregulated or downregulated by RNA-Seq in BAL cells of healthy volunteers were chosen for confirmation by quantitative real-time PCR in the same samples used for RNA-Seq analysis (Fig 6). We confirmed upregulation of *CXCL11*, *CXCL10*, *IL10*, *USP41*, *USP18*, and *IRF7* and downregulation of *ADAMTS15*, *HPGD*, *LRP1*, and *CD36*. A subset of differentially regulated genes identified from the RNA-Seq data was also selected for validation in a second independent cohort of volunteers (Fig 7). The increased expression of *USP41* and *IL10* were confirmed using real-time PCR in this cohort, as well as the downregulation of *ADAM15* and *LRP1* (CD91).

## Discussion

The ability to sense and respond to a wide array of microbial signals and activate the innate immune program is a vital function of resident macrophages within the airspaces. While transmembrane TLR survey the extracellular space and PRRs such as RIG-I family, NOD-like receptor proteins, and DNA nucleic acid sensors survey the cytosol, accumulating evidence indicates that these PRRs converge upon a type I IFN transcriptional program [36]. Type I IFN response was initially identified as an essential component of host anti-viral immunity in vertebrates. However, recent studies indicate that the type I interferons exert a more complex, dual role comprised of both a stimulatory as well as immune regulatory function [38, 47]. This dual response may be a physiologic response that is hard-wired in the healthy alveolar macrophage, as a delicate balance of both positive and negative regulatory signals are required to eliminate offending agents yet simultaneously prevent excessive inflammation and injury [48]. Indeed, monogenic disorders such as Aicardi-Goutières syndrome and type I interferonopathies bring attention to the tight regulation required to control type I interferon response and prevent auto-inflammatory diseases in humans [49].

In this report, we utilized whole transcriptomic profiling through RNA-Seq to assess the full spectrum of LPS-induced TLR4 activation in alveolar macrophages from healthy volunteers in vitro. We show a prominent type I interferon response following TLR4 activation with induction of *TICAM1*, *IRF7*, *IFNB*, *IFNAR*, and *ISGs*. TRIF (TICAM1) is critical for upregulation of costimulatory molecule expression following TLR4 activation [50] and this pathway requires autocrine and paracrine stimulation through IFNAR [51]. TRIF also signals the type I interferon pathway through which downstream caspase 11 activation mediates NLRP3-dependent caspase 1 activation and IL-1β processing [10]. In human alveolar macrophages, gene expression of *CASP4* and *CASP5*, human homologues of murine caspase 11 [52], as well as of *CASP1* and *NLRP3* is increased following stimulation with ultrapure LPS. We also observe concomitant activation of *USP-18* and *ISG-15*, negative regulators of type I interferon signaling, at the onset of LPS-stimulated responses. In addition, among the top upregulated genes are *IRG1* and *IDO1* that encode enzymes controlling cellular metabolism, curtail NFκB-mediated inflammation [30] and promote endotoxin tolerance [31].

IRF-7 was identified as a central transcriptional factor upstream of 89 known gene targets that were induced upon LPS stimulation. The role of IRF-7 and IRF-3 in inflammation has been extensively studied in viral responses, as they control the expression of type I interferons. Evidence indicates that IRF-3 and IRF-7 are also activated upon TLR4 binding of LPS via TRIF activation, independent of MYD88 [38]. Several of the IRF-7 targets expressed in our dataset are known to be negative regulators of inflammation, including *USP18*, *ISG15*, *TREX1*, and *ADAR*. We also identified a novel *USP41* gene with predicted sequence similar to that of *USP18*, a critical negative regulator of type I IFNs, as a top upregulated gene induced by LPS. *USP41* was also clustered by expression level close to *USP18* and other type I interferon related genes. To the best of our knowledge, this is the first report showing induction of *USP41* gene in alveolar macrophages after LPS stimulation.

The findings of our study are in line with recent reports using microarrays-based expression profiling of murine macrophages, where upon LPS stimulation, type I interferon related genes, including *IRF7* are upregulated. This group of genes is also part of a core macrophage response module to different stimuli comprising several TLR activators and innate particles [53, 54]. Interestingly, a similar number of IRF-7 target genes, approximating 80 were upregulated in one of these studies in murine macrophages [54]. In addition, a recent report using RNA sequencing in human macrophages derived from the monocytic THP-1 cell line infected with the gram negative bacteria *Campilobacter concisus* showed that type I interferon related genes were the top three upregulated category, including strong induction of *IRF7* [55]. The most comprehensive transcriptomic analysis in human macrophages is a report by Xue et al.[1] examining the response of human monocyte-derived macrophages to a wide variety of stimuli including LPS. Interestingly, *IRF7* and *IL10* were highly expressed in human monocyte derived macrophages stimulated with LPS in their study. However, a detailed pathway analysis within the LPS stimulated cells is not available to draw further comparisons to our study. Our study is the first reporting in detail the transcriptomic response to endotoxin in primary human alveolar macrophages, demonstrating cross activation of TLR4, RIG-I and NOD-like receptor proteins, DNA nucleic acid sensors, and IRF-7 as the top upstream regulator.

IL-10 limits immune response to pathogen but over-vigorous IL-10 production early during microbial infection can be detrimental to the host [40]. As *IL10* was among the top 50 upregulated genes and was closely clustered to other IRF-7 target genes in the heat map, we evaluated the role of IRF-7 in LPS-induced IL-10 production by transfecting HMDM with IRF-7 siRNA and observed enhanced production of IL-10. Others have shown the requirement for type I interferon signaling in LPS-induced IL-10 production in mouse bone-marrow derived macrophages through TRIF, IRF-3 and IFNAR [39]. In contrast to this finding but consistent with our study, murine *Irf7*^*-/-*^ conventional CD11c^hi^ splenic dendritic cells exhibit enhanced LPS-induced IL-10 production in vitro that reduce their ability to drive Th1 responses [56]. Thus, it appears that discrete components of type I interferon signaling such as IRF-7 play dual roles by finely calibrating both stimulatory and inhibitory signals to modulate host cytokine response to endotoxin challenge. Supporting this hypothesis, we show that USP-18, a target of IRF-7 and known negative regulator of the type I interferon response, decreases the production of immune-regulatory cytokines IL-27 and IL-10. We speculate mechanisms to curtail over-vigorous anti-inflammatory cytokine production early during pathogen challenge may be beneficial to host.

Compound heterozygous mutations in IRF-7 leads to reduced type I interferon responses in otherwise healthy children, and is associated with the development of severe life threatening consequences of influenza infection such as acute respiratory distress syndrome [57]. In addition, it is well documented that patients who develop influenza are prone to secondary gram negative and gram positive bacterial infections that contribute to morbidity and mortality [58]. Type I interferons, which are essential for antiviral immunity, can impair subsequent host response to bacterial challenge following influenza infection in mice [59]. Our findings highlight a potential novel role by which IRF-7 can restrain early IL-10 responses known to be detrimental to anti-bacterial host defense.

One limitation of our study is the use of single-time point of LPS stimulation. However, previous studies in murine macrophages using microarrays have shown that most of the transcription factors upregulated by LPS and their target genes are activated between 2–4 hours and remain in this state at 6–8 hours [54]. Moreover, our findings were validated in an independent cohort in which LPS was used to stimulate macrophages for 2 hours. The limited amount of primary alveolar macrophages obtained from the healthy volunteers precluded performing formal time course experiments; however, we were able to capture the most informative time points.

In summary, human alveolar macrophages in health calibrate LPS-induced responses by mounting a prominent type I interferon transcriptional signature with induction of *IRF7*. We show concomitant induction of *USP18*, a negative regulator of interferon-α/β responses, previously implicated in controlling human auto-inflammatory disease [33]. Moreover, our findings indicate that *IRF-7* and *USP18* calibrate downstream macrophage effector cytokine production such as IL-10. Our studies provide the framework for future studies examining human alveolar macrophage transcriptional responses in disease cohorts.

## Supporting Information

S1 Table Top 100 down-regulated genes (control vs. LPS, t-test, adjusted *p-value <0*.*05*, Benjamini-Hochberg). Values represent mean expression (log2).
(DOCX)

S2 Table Top 100 up-regulated genes (control vs. LPS, t-test, adjusted *p-value <0*.*05*, Benjamini-Hochberg). Values represent mean expression (log2).
(DOCX)

S1 Appendix Expression values of all downregulated genes. (control vs. LPS, t-test, adjusted *p-value <0*.*05*, Benjamini-Hochberg). Values represent mean expression (log2).
(XLSX)

S2 Appendix Expression values of all upregulated genes. (control vs. LPS, t-test, adjusted *p-value <0*.*05*, Benjamini-Hochberg). Values represent mean expression (log2).
(XLSX)
