# Supplementary material for: Full Spectrum of LPS Activation in Alveolar Macrophages of Healthy Volunteers by Whole Transcriptomic Profiling
Source: PLoS One. 2016 Jul 19;11(7):e0159329. doi: 10.1371/journal.pone.0159329 (PMC4951018; doi:10.1371/journal.pone.0159329)
Supplement: S1 Table — Values represent mean expression (log2). (DOCX) [file pone.0159329.s001.docx]

| **Gene** | **Control** | **LPS** | **Gene** | **Control** | **LPS** |
| --- | --- | --- | --- | --- | --- |
| ADCY1 | 2.21341 | -1.84775 | AOX1 | 0.394751 | -2.54558 |
| CYP1A1 | 2.16601 | -1.8812 | HAL | 0.172636 | -1.9642 |
| IQGAP3 | 5.14122 | 1.20823 | Cyclin-dependent kinase-like 3 | 0.459256 | -1.65899 |
| TMEM163 | 3.22379 | -0.302717 | UMODL1 | 2.21986 | 0.103482 |
| METTL7B | 0.794861 | -2.7116 | DOCK3 | 3.18569 | 1.07941 |
| C2orf71 | 1.93988 | -1.5108 | PPARGC1B | 2.87356 | 0.775163 |
| SLIT3 | 1.73157 | -1.60273 | KIAA1737 | 4.9803 | 2.88262 |
| ADAMTS15 | 4.14836 | 0.893044 | ADFP | 11.254 | 9.16028 |
| SPTBN4 | 2.30905 | -0.933118 | RRM2 | 2.40982 | 0.344661 |
| HPGD | 6.59849 | 3.39749 | RAB3D | 0.210713 | -1.85304 |
| UCN2 | 0.651073 | -2.40997 | THBD | 9.73781 | 7.71011 |
| PCSK6 | 0.066232 | -2.97598 | SHB | 4.7728 | 2.75176 |
| PDGFRB | -0.597525 | -3.46257 | RAB3IL1 | 0.060783 | -1.95013 |
| Similar to hCG2014363 | 1.15149 | -1.62859 | GPRIN3 | 6.61106 | 4.60059 |
| AGPAT9 | 6.75238 | 3.97341 | Kazrin | 4.44157 | 2.43199 |
| TBC1D2 | 7.39392 | 4.68664 | EPB41L1 | 7.1302 | 5.12415 |
| C4orf18 | 1.59052 | -1.10975 | PAQR5 | 7.07897 | 5.07358 |
| HES2 | 4.91901 | 2.29749 | HSD3B7 | 6.71889 | 4.71553 |
| EEPD1 | 2.66983 | 0.077376 | TXNIP | 9.22201 | 7.22196 |
| CORO2A | 7.59635 | 5.06014 | Hypothetical protein LOC100507386 | 0.446313 | -1.55242 |
| GFOD1 | 4.11584 | 1.60291 | HIC1 | 1.34002 | -0.656992 |
| UBASH3B | 6.60372 | 4.09163 | Hypothetical LOC100507360 | 0.669651 | -1.32703 |
| LOC284837 | 5.11233 | 2.61031 | C10orf128 | 4.85018 | 2.85684 |
| ARHGAP6 | 3.15977 | 0.66069 | RHOBTB2 | 4.84448 | 2.86242 |
| Hypothetical LOC100507642 | 0.456836 | -2.02859 | CABLES1 | 4.30589 | 2.32427 |
| HAVCR1 | 0.023149 | -2.4485 | PLA2G15 | 6.85572 | 4.88331 |
| VWF | 1.99905 | -0.461821 | CLCN4 | 3.22035 | 1.24862 |
| LOC100132526 | 0.89603 | -1.5367 | Tctex1domain containing 2 | 2.96152 | 0.994914 |
| ZNF788 | 2.39561 | -0.036154 | PIK3CG | 5.10225 | 3.14197 |
| ZNF589 | 5.92656 | 3.52613 | PFKFB2 | 3.74365 | 1.78941 |
| SLC29A3 | 4.48366 | 2.14105 | COL15A1 | -0.179377 | -2.1315 |
| AKAP1 | 4.57331 | 2.23231 | LRRC25 | 3.28581 | 1.3415 |
| OLFML2B | 1.00187 | -1.30565 | FABP3 | 8.65268 | 6.70921 |
| LOC440028 | 1.21737 | -1.08702 | TNFSF15 | 5.08517 | 3.1503 |
| TTYH3 | 6.54354 | 4.23953 | TPD52L1 | 2.31284 | 0.378689 |
| Hypothetical protein LOC100506752 | 0.783924 | -1.50801 | CD180 | 1.26732 | -0.653695 |
| SCG5 | 1.75563 | -0.519905 | AURKA | 1.65916 | -0.258239 |
| CEP55 | 1.4342 | -0.840164 | KIF16B | 6.10975 | 4.19764 |
| GAS2L3 | 5.10867 | 2.84699 | CD36 | 6.39257 | 4.48109 |
| SHANK3 | 0.708191 | -1.55013 | CLMN | 4.78766 | 2.89345 |
| ABCG1 | 8.17851 | 5.93299 | S100A16 | 4.10211 | 2.21403 |
| SEMA6B | 5.60357 | 3.35984 | THRA | 5.38533 | 3.49764 |
| C6orf114 | 2.16192 | -0.077834 | FGD4 | 4.23861 | 2.36616 |
| COL4A2 | 1.96284 | -0.270772 | GPR34 | 2.24716 | 0.375206 |
| PRKAG3 | 0.969423 | -1.261 | AADACL1 | 7.50674 | 5.63946 |
| CRTAM | 5.06669 | 2.8553 | MOSPD1 | 5.47981 | 3.61255 |
| ST6GALNAC4 | 6.42279 | 4.22311 | ANKRD29 | 1.43657 | -0.427015 |
| KCNAB2 | 7.14851 | 4.97374 | TOP2A | 2.22045 | 0.360562 |
| KIAA1045 | -0.12677 | -2.3011 | ZMIZ1 | 7.80695 | 5.96388 |
| KRT79 | 3.57535 | 1.42084 | LDLRAP1 | 5.33038 | 3.48903 |

**S1 Table.** Top 100 down-regulated genes (control vs. LPS, t-test, adjusted *p-value <0.05*, Benjamini-Hochberg). Values represent mean expression (log2).
