# Supplementary material for: Full Spectrum of LPS Activation in Alveolar Macrophages of Healthy Volunteers by Whole Transcriptomic Profiling
Source: PLoS One. 2016 Jul 19;11(7):e0159329. doi: 10.1371/journal.pone.0159329 (PMC4951018; doi:10.1371/journal.pone.0159329)
Supplement: S2 Table — Values represent mean expression (log2). (DOCX) [file pone.0159329.s002.docx]

| **Gene** | **Control** | **LPS** | **Gene** | **Control** | **LPS** |
| --- | --- | --- | --- | --- | --- |
| CXCL11 | 0.072935 | 6.83612 | NT5C3 | 4.18222 | 7.95444 |
| PDGFRL | -2.975 | 3.15402 | IL23A | 4.09881 | 7.85441 |
| NEXN | 1.07392 | 6.75192 | CCL4 | 8.57772 | 12.3316 |
| IRG1 | 3.50984 | 8.89334 | LRRC50 | 0.545814 | 4.26661 |
| USP41 | 0.925952 | 6.11949 | Hypothetical gene LOC440896 | 2.32173 | 6.00582 |
| ISG20 | 3.08171 | 8.23432 | SPINK7 | -1.61224 | 1.99307 |
| CXCL10 | 3.07556 | 8.21614 | ZBTB32 | -1.87344 | 1.72355 |
| IL27 | -0.563753 | 4.53168 | NCRNA00158 | -1.06732 | 2.49491 |
| USP18 | 2.5383 | 7.60631 | Hypothetical LOC100507056 | -2.75638 | 0.783425 |
| TMEM171 | 0.082037 | 5.05975 | TNFSF10 | 2.32313 | 5.84822 |
| CSF3 | 2.93409 | 7.89531 | MX2 | 4.92219 | 8.417 |
| IFIT1 | 4.32571 | 9.22816 | IGF2BP3 | -1.04913 | 2.42317 |
| CSF2 | 2.03051 | 6.8378 | ZNF618 | -0.706792 | 2.75731 |
| ETV7 | -0.175017 | 4.59096 | HSH2D | 1.41235 | 4.8665 |
| IL1F9 | -0.071786 | 4.67239 | CFB | 4.79282 | 8.24361 |
| IL6 | 6.36476 | 10.9948 | OAS2 | 6.70836 | 10.155 |
| SSTR2 | -0.006047 | 4.55477 | GCKR | -1.36503 | 2.05523 |
| IL15RA | 3.32955 | 7.88693 | DDX58 | 5.28936 | 8.65447 |
| IFIT3 | 6.32483 | 10.8609 | SAMD9 | 5.88256 | 9.24443 |
| BCL2L14 | -3.23939 | 1.26691 | C14orf83 | 0.763 | 4.11868 |
| CCL19 | -3.95638 | 0.511703 | TSLP | 2.62504 | 5.97922 |
| IFIT2 | 5.71344 | 10.1788 | EPSTI1 | 4.41921 | 7.73524 |
| SERPINB7 | -1.57142 | 2.77782 | DPP4 | 2.37913 | 5.66962 |
| IL19 | -2.775 | 1.54462 | PNPT1 | 4.04646 | 7.33084 |
| PRIC285 | 5.80048 | 10.0985 | C7orf51 | -3.09199 | 0.101562 |
| IFIT1L | -0.24782 | 4.0307 | MX1 | 7.24161 | 10.428 |
| CKB | 3.28952 | 7.56263 | GBP7 | 2.08654 | 5.23268 |
| IDO1 | 3.29972 | 7.48862 | EXT1 | 2.83239 | 5.97565 |
| ISG15 | 5.72065 | 9.86028 | IL2RA | 1.72419 | 4.8466 |
| CCL4L1 | 7.28778 | 11.4272 | CCL3 | 9.64991 | 12.7316 |
| BATF2 | 1.93633 | 6.02145 | IFIH1 | 6.05338 | 9.13104 |
| Hypothetical LOC100507575 | -3.12239 | 0.949423 | IL12A | -2.65801 | 0.412532 |
| CCL4L2 | 6.94274 | 11.0104 | OAS3 | 7.26592 | 10.331 |
| GBP4 | 4.30536 | 8.35982 | C15orf26 | -2.08841 | 0.929525 |
| Hypothetical LOC541472 | -2.92239 | 1.11959 | GBP5 | 4.97061 | 7.97895 |
| HESX1 | -2.72239 | 1.2885 | KLK10 | -0.859136 | 2.14902 |
| RTP4 | 0.981956 | 4.98705 | ANKRD1 | -0.260886 | 2.73301 |
| IFI44 | 4.81608 | 8.81855 | CYP7B1 | -2.92239 | 0.051356 |
| IL10 | -0.234161 | 3.72243 | SLAMF1 | 2.648 | 5.61152 |
| IFNB1 | -2.85801 | 1.07753 | IRF7 | 5.60503 | 8.56175 |
| HERC5 | 4.36516 | 8.27361 | IL1B | 9.92289 | 12.8724 |
| CCL3L1 | 7.15942 | 11.0285 | HHLA2 | 1.17119 | 4.11859 |
| PLEKHA4 | 1.95586 | 5.81726 | SLC38A5 | -0.755262 | 2.18452 |
| RANBP3L | -1.90466 | 1.94896 | IL12B | -3.15638 | -0.220613 |
| HERC6 | 3.78433 | 7.63115 | KCTD14 | -2.83939 | 0.092976 |
| CCL3L3 | 7.20689 | 11.0385 | TNF | 8.9283 | 11.8343 |
| Chloride channel accessory 3 (pseudogene) | -3.75638 | 0.072474 | ADORA2A | 3.75631 | 6.66026 |
| DHX58 | 3.54219 | 7.36842 | XAF1 | 5.35272 | 8.254 |
| IFI44L | 5.03944 | 8.86212 | PML | 5.41116 | 8.30632 |
| ZBP1 | 0.909538 | 4.69272 | IFI35 | 5.02742 | 7.91751 |

**S2 Table.** Top 100 up-regulated genes (control vs. LPS, t-test, adjusted *p-value <0.05*, Benjamini-Hochberg). Values represent mean expression (log2).
